# Management of Childhood Headache in the Emergency Department. Review of the Literature

**DOI:** 10.3389/fneur.2019.00886

**Published:** 2019-08-23

**Authors:** Umberto Raucci, Nicoletta Della Vecchia, Chiara Ossella, Maria Chiara Paolino, Maria Pia Villa, Antonino Reale, Pasquale Parisi

**Affiliations:** ^1^Pediatric Emergency Department, Bambino Gesù Children's Hospital, IRCCS, Rome, Italy; ^2^Department of Pediatrics, University of “Studi della Campania Luigi Vanvitelli”, Naples, Italy; ^3^Chair of Pediatrics, NESMOS Department, Faculty of Medicine and Psychology, Sapienza University, Sant' Andrea Hospital, Rome, Italy

**Keywords:** headache, migraine, emergency, child, life threatening condition, secondary headache, diagnosis, neuroimaging

## Abstract

Headache is the third cause of visits to pediatric emergency departments (ED). According to a systematic review, headaches in children evaluated in the ED are primarily due to benign conditions that tend to be self-limiting or resolve with appropriate pharmacological treatment. The more frequent causes of non-traumatic headache in the ED include primitive headaches (21.8–66.3%) and benign secondary headaches (35.4–63.2%), whereas potentially life-threatening (LT) secondary headaches are less frequent (2–15.3%). Worrying conditions include brain tumors, central nervous system infections, dysfunction of ventriculo-peritoneal shunts, hydrocephalus, idiopathic intracranial hypertension, and intracranial hemorrhage. In the emergency setting, the main goal is to intercept potentially LT conditions that require immediate medical attention. The initial assessment begins with an in-depth, appropriate history followed by a complete, oriented physical and neurological examination. The literature describes the following red flags requiring further investigation (for example neuroimaging) for recognition of LT conditions: abnormal neurological examination; atypical presentation of headaches: subjective vertigo, intractable vomiting or headaches that wake the child from sleep; recent and progressive severe headache (<6 months); age of the child <6 years; no family history for migraine or primary headache; occipital headache; change of headache; new headache in an immunocompromised child; first or worst headache; symptoms and signs of systemic disease; headaches associated with changes in mental status or focal neurological disorders. In evaluating a child or adolescent who is being treated for headache, physicians should consider using appropriate diagnostic tests. Diagnostic tests are varied, and include routine laboratory analysis, cerebral spinal fluid examination, electroencephalography, and computerized tomography or magnetic resonance neuroimaging. The management of headache in the ED depends on the patient's general conditions and the presumable cause of the headache. There are few randomized, controlled trials on pharmacological treatment of headache in the pediatric population. Only ibuprofen and sumatriptan are significantly more effective than placebo in determining headache relief.

## Introduction

Headache is a frequent complaint in pediatric population, even more frequent than adults. There has been a substantial increase in the incidence of childhood migraine and headache over the last 30 years. This increase is alarming and likely reflects children's lifestyles (1). Diagnosis and treatment can be challenging due to its varying presentation, etiology, and triggers. Secondary headaches manifest differently in children than in adults and the degree of brain maturation could be the cause of this difference (2, 3). Headache disorders are the main cause of absence from school, affecting negatively school performance (4), as well as other daily activities (5). Headaches are common, incapacitating, and often stress inducing for pediatric patients and parents alike. The individual and social costs of pediatric headache disorders are due to the high incidence, frequency, and lifetime prevalence of these conditions (6).

The incidence of headache is variable according with age (3–8% of children aged 3 years, 19.5% of children aged 5, and 37–51.5% of children aged 7) (7–9). Frequency is higher in males before puberty and in females after puberty (10). Another important consideration is that 35% of children with headache present to an emergency department (ED) at least once a year for any reason, compared with 17% of the general pediatric population (11). Children suffering from headaches also have a higher rate of hospitalization for any reason (5.1% per year) compared to children without headaches (1.7% per year) (12). Non-traumatic headaches represent 0.7% (13) to 2.6% (11) of visits in a pediatric ED. The hospital admission rate for headache ranges from 8 (12) to 29% (14) in studies carried out in patients accessing the ED. Headache is unusual as an isolated complaint and is most often associated with other symptoms such as fever, sore throat, neck pain, and vomiting (15). The most common recurrent headache in childhood is migraine, while tension headaches prevail in adolescence (16). Males are more frequently affected at preschool age, while incidence is higher in females in junior-high school age (17, 18). Differential diagnosis of pediatric headache in the ED includes a variety of benign causes and viral infections, sinusitis, migraine, and post-traumatic headaches are the most common diagnoses (2). Burton et al. (13) reported viral infections, sinusitis and pharyngitis in over 60% of pediatric patients presenting to the ED for headache. Secondary life-threatening (LT) causes of headache can be associated with high mortality and morbidity and health personnel should be aware of the differential diagnoses. Headache in the ED is mainly due to benign conditions that tend to be self-limiting or resolve after appropriate treatment. The most common causes of non-traumatic headache in the ED are secondary benign headaches (35.4–63.2%) and primary headaches (21.8–66.3%) and while secondary LT headaches are less frequent (2–15.3%; Table 1) (11, 13, 18–26). Conditions to worry about include brain tumors, central nervous system infections, ventriculo-peritoneal shunt malfunction, hydrocephalus, idiopathic intracranial hypertension and intracranial hemorrhage (Table 2) (3, 13, 27).

**Table 1:** Etiology of headache in Emergency Department: comparison of the published studies.

	**Burton LJ** **(11)**	**Kan L** **(19)**	**Lewis DW** **(20)**	**Leon-Diaz A** **(21)**	**Conicella E** **(14)**	**Scagni P** **(22)**	**Lateef TM** **(23)**	**Hsiao HJ** **(24)**	**Massano D** **^**a**^ (11)**	**Rossi R** **(25)**
Years of publication Years of Recruitment	1997 1993	2000 1996	2000 1996	2004 2002–2003	2008 2004	2008 2003–2004	2009 2003–2006	2014 2008	2014 2009–2012	2018 2011–2015
Number of patients	696	130	150	185	432	526	364	409	101	1,833
Patients Age(years) (mean–ag.e years)	2–18	<18 (9.3)	<IS (9)	2–15	2–17 (8.9)	0–16 (8.8)	2–5	2.6–17.8 (9.2)	6–18	<18 (9.68)
Percentage (%) of ED visits	1.3	0.7	^ne^	0.57	0.8	1.0	ne	0.9	2.63	0.9
Primary headaches	21.8	10	18	24.3	24.5	56.7	15.7	27.6	66.3	62.1
Secondary benign headaches	63.2	63.2	59.6	60.5	35.4	38	72.3	65.6	33%	32.9
Secondary life-threatening headaches Brain Tumors %	5.6 ne	15.3 1.5	14.9 2.6	4.3 2.5	4.1 0.69	4 0.36	7.9 0.2%	6.8 0.97	9.9 1.9	1.3 0.38
Unclassified	13	11.5	7	10.8	36	1.3	5	5	ne	7.8

ED, Emergency Department; ne, not expressed;

a Only patients with focal neurological signs at admission to ED.

**Table 2:** Life-threatening causes of headache in children.

Hypertension Coarctation of aorta Central nervous system infections Ventriculo-peritoneal shunt malfunction or infection Venous sinus thrombosis Ischaemic Stroke Reversible cerebral vasoconstriction syndrome (RCVS) Cervical artery dissection Hemorrhage Brain tumor Hydrocephalus Brain malformation (Chiari type I, Dandy Walker) Idiopathic intracranial hypertension Carbon monoxide poisoning

The following work aims to suggest useful elements for the ED pediatrician in the management of headaches in children. In particular, the identification of factors associated with LT secondary headache (red flags), the identification of causes of LT headaches and the rational use of laboratory tests and diagnostic imaging are discussed.

### Headache Classification

The International Headache Society (IHS) (28) publishes a standardized classification scheme that provides diagnostic criteria for headaches in general and its most recent update was released in 2018 (Table 3).

**Table 3:** ICHD-3 revised Headache Classification (28).

**Primary headache** Migraine (with or without aura) Tension-type headache Trigeminal autonomic cephalalgias Other primary headache disorders **Secondary headache** Headache attributed to trauma or injury to the head and/or neck Headache attributed to cranial and/or cervical vascular disorder Headache attributed to non-vascular intracranial disorder Headache attributed to a substance or its withdrawal Headache attributed to infection Headache attributed to disorder of homeostasis Headache or facial pain attributed to disorder of the cranium, neck, eyes, ears, nose, sinuses, teeth, mouth, or other facial or cervical structure Headache attributed to psychiatric disorder **Painful cranial neuropathies, other facial pain, and other headaches** Painful lesions of the cranial nerves and other facial pain Other headache disorders.

## Primary Headache

### Migrane Without Aura

Primary headache accounts for 21.8–66.3% of headaches in children and migraine is the most frequent type. Migraine is a recurrent headache disorder that manifests with attacks lasting 4–72 h (28) (Tables 4, 5). In children up to 5 years of age, a shorter duration period for the attacks has been suggested (29). The pain is typically unilateral, pulsating, of moderate or severe intensity, aggravated by physical activity and associated with nausea and/or photophobia and phonophobia (28).

**Table 4:** ICHD-3 diagnostic criteria for migraine without aura (28).

**A.** At least five attacks fulfilling criteria B-D **B.** Headache attacks lasting 4–72 h (when untreated or unsuccessfully treated) **C.** Headache has at least two of the following four characteristics: 1. Unilateral location 2. Pulsating quality 3. Moderate or severe pain intensity 4. Aggravation by or causing avoidance of routine physical activity (e.g., walking or climbing stairs) **D.** During headache at least one of the following: 1. Nausea and/or vomiting 2. Photophobia and phonophobia **E.** Not better accounted for by another ICHD-3 diagnosis.

**Table 5:** ICHD-3 diagnostic criteria for migraine with aura (28).

**A.** At least two attacks fulfilling criteria B and C **B.** One or more of the following fully reversible aura symptoms: 1. Visual 2. Sensory 3. Speech and/or language 4. Motor 5. Brainstem 6. Retinal **C.** At least three of the following six characteristics: 1. At least one aura symptom spreads gradually over ≥ 5 min 2. Two or more aura symptoms occur in succession 3. Each individual aura symptoms lasts 5–60 min 4. At least one aura symptom is unilateral 5. At least one aura symptom is positive 6. The aura is accompanied, or followed within 60 min, by headache **D.** Not better accounted for by another ICHD-3 diagnosis.

The prevalence ranges from 3.2 to 14.5%. Family history is often positive for headache with a frequency of 60–77.5% (3). As already observed by other authors we believe that the time span of headache attacks in children should be changed to 30 min or longer. This could result in a greater number of children being diagnosed with migraine, in particular those younger in age (3, 30). In children, pain is more frequently frontal (60.9%), whereas it is ocular (53.17%) followed by temporal (38.67%) in adults (31). Pain is usually described by children as throbbing or pounding, while it is frequently pulsating in adults (32). It is common practice that when the episodes are specific for duration and characteristics, the diagnosis of migraine can be made before five episodes. In the new revision of the ICHD-3 (28) five episodes of headache are still necessary for diagnosing migraine. In an emergency setting this seems to be limitation and some authors (30) have proposed reducing the number of episodes needed for diagnosing migraine.

The typical headache pattern and associated symptoms make it possible to differentiate migraine without aura from other forms of primary and secondary headache.

In the case of headache with features highly suggestive of migraine, a completely negative neurological examination and the absence of so-called “red flags” suggest that the patient can be sent to a specialized Headache Center. In an observational study, other authors report a reduction in ED access for recurrent headache in those patients for whom indication was given to contact a specialized Headache Center within 10 days from ED discharge (22).

### Migrane With Aura

Migraine with aura is characterized by transitory focal neurological symptoms that generally appear before or sometimes together with the cephalalgic pain. A prodromal phase may be present in some patients, which occurs hours or days before the onset of headache/or a post-dromal phase that appears after the resolution of the headache. Symptoms include hyperactivity, hypoactivity, depression, cravings for certain foods, repetitive yawning, fatigue and stiffness, and/or neck pain (28). Visual and sensory auras are the more common symptoms (87.1%) in pediatric population as well as in adults. Migraine with aura is most common in adolescents compared to younger children (3), but this may be due to the inability of young children to describe their symptoms clearly (33).

Migraine with brainstem aura is a particular kind of migraine in which symptoms of aura originate unequivocally from the trunk-encephalic region and/or reflect the simultaneous involvement of both hemispheres, in the absence of motor deficits (28). The symptoms of aura of this particular form of migraine are immediately traceable to the brain stem in the absence of an ischemic etiology. This condition consists of completely reversible words/language, sensory or visual auras with retinal symptoms or lasting engines, by definition, from 5 to 60 min. A cephalalgic pain may accompany the aura within 1 h. The most common symptoms are nausea and vomiting (30–50%), ataxia (43–50%), bilateral visual symptoms, or altered consciousness. In these cases, posterior fossa circulatory insufficiency (vertebral dissection or thrombosis), transient ischemic attack, posterior fossa vascular, and congenital structural abnormalities may need to be excluded by MRI (34). The difference between migraine with brainstem aura and migraine with typical aura is the origin of the symptoms. In the first case the brainstem or bilateral occipital hemispheres are involved, while migraine with typical aura is mainly restricted to a unilateral hemisphere.

Hemiplegic migraine (HM) is a type of migraine with aura and motor weakness. For diagnosis, fully reversible motor weakness is associated with constant aura symptoms, consisting in visual, sensory, and/or speech/language disorders (28). However, more than 70% of patients have baseline symptoms with prolonged hemiplegia, confusion, coma, fever, or seizures. In this case it is imperative to exclude an acute ischemic process by performing brain MRI with DWI sequences.

It is mandatory in the ED setting to exclude those secondary headache disorders than can mimic migraine and are potentially life threatening (35, 36), by investigating for red flags (Table 6). Furthermore, it is essential to know the characteristics of the primary forms of migraine in order to avoid unnecessary, expensive, and potentially dangerous investigations (for example neuroradiological imaging**)**. Nevertheless, it is clear that whenever there is suspicion for secondary headache, migraine should be a diagnosis of exclusion. Migraine is also the more common cause of brain attack (stroke-like) symptoms in children accessed to the ED, accounting for 11–29% of cases (37). Some warning signs can significantly raise the suspicion of a secondary form due to stroke or other vasculopathies: rapid onset of headache, presence of focal neurological signs and/or symptoms and altered consciousness. In these cases it is necessary to carry out neuroimaging studies. This is particularly relevant for a thunderclap headache (more frequent in adults), which warrants rigorous evaluation to exclude a secondary cause. Any headache with a very rapid onset reaching peak intensity in <1 min is, by definition, a headache of thunderclap onset and can be a symptom of a subarachnoid hemorrhage, hemorrhagic stroke, reversible cerebral vasoconstriction syndrome, venous sinus thrombosis, or even pituitary apoplexy (38). The more gradual onset of neurological symptoms in migraineurs, usually >5 min, is attributed to the cortical spreading depression of Leao, consisting of depolarization followed by hyperpolarization, at a speed of 3–5 mm per min, across the cerebral cortex (39). Furthermore, hemiparesis is an infrequent form of migraine aura, with a frequency of <10% and seldomly presents without other symptoms. In sporadic hemiplegic migraine, motor symptoms develop gradually over minutes, affecting more often the arm than the leg (while sparing the face). They can be bilateral and are primarily associated with headache (40). Another useful element is that children with arterial ischemic stroke (AIS) are older than those with migraine, and considered at risk (red flag) up to 8 years of age (40). In fact, migraine with aura is uncommon in children <8 years of age and has a prevalence of 3–4% between age 3–7 years, compared to 23–31% in teenagers (11, 41). Odds of AIS are significantly increased in the case of sudden onset of the following symptoms: weakness, seizures, speech disturbance, ataxia, signs of face, inability to walk, dysarthria, dysphasia, and altered consciousness. Significant features associated with decreased odds of AIS include older age, vomiting, visual or sensory aura, other symptoms, and absent focal signs on assessment (40). Hypersensitivity conditions such as photophobia, phonophobia, or osmophobia are often seen only with migraine. Visual aura, in the form of positive signs such as zigzag lines or spreading scintillating scotoma, is by far the most common, unilateral sensory disturbance. Dysphasia may occur either at the same time or sequentially. Sometimes aura may occur without headache and must be differentiated from stroke in which negative signs with visual defects are present. Furthermore, a migrainous aura typically develops within a few minutes and migrates from one area to another (36, 42, 43).

**Table 6:** Warning signs in children with headache (red flags).

**Red flags**
Changes in mood or personality over days or weeks Related to severe vomiting, especially in early morning Worsening of pain with cough or Valsalva maneuverAltered conscious state Papilledema Focal neurologic deficit or meningismus Seizures or fever High-risk population (patients with sickle cell anemia., malignancy, recent head trauma, ventricular-peritoneal shunt, others) Pain that wakes the c.hild from sleep or occurs on waking Change of the character of headache in patients diagnosed with primary headache Poor general condition Increased head circumference Cranial nerve palsies Abnormal ocular movements, squint, pathologic pupillary responses Visual field defects Ataxia, gait abnormalities, impaired coordination Sudden onset of headache (first or worst ever) Increase in severity or characteristics of the headache Occipital headache^*^ Age < 5 years^*^

Modified by Roser et al. (36);

* relative red flags.

### Episodic Syndromes That May be Associated With Migraine

The “childhood periodic syndromes” were renamed by the ICHD-3 as the “episodic syndromes that may be associated with migraine” (44) and include four main conditions: two recurrent gastrointestinal disorders: cyclic vomiting syndrome and abdominal migraine; benign paroxysmal vertigo; benign paroxysmal torticollis. Common features of these disorders are: complete well-being between episodes, stereotypy of episodes, familiarity for migraine or headache (2). Patients with this group of disorders can present with migraine (with or without aura) or are likely to develop them. These patients may also have motion sickness or periodic sleep disorders (28). Episodic syndromes can be difficult to recognize and treat. These patients often undergo intensive diagnostic workup, including neuroimaging, and frequently require access to ED as well as hospital admissions.

*Recurrent gastrointestinal* disorders are defined as episodes of recurrent abdominal pain and/or discomfort, nausea, and/or vomiting, which occur rarely, chronically or at foreseeable intervals. These conditions can be associated with migraine (28). An acute presentation could be confused with an acute abdomen or an intracranial mass, though a detailed history and physical examination should rule them out. A more subtle presentation could suggest a systemic disease that may take time to evolve (44).

*Cyclic vomiting syndrome* is defined as episodes of intense nausea and vomiting, usually stereotypical, with predictable timing of the episodes. Attacks may be associated with pallor and lethargy. A typical element is the complete resolution of symptoms between attacks. Nausea and vomiting occur at least four times per hour and the attacks last for 1 h, up to 10 days and occur 1 week apart (28). Symptoms can be so intense as to require ED management (45) and hospitalization for intravenous rehydration (44). The diagnosis is generally delayed and these patients often undergo multiple hospitalizations and invasive diagnostic tests. The differential diagnosis is made with acute abdominal disease, intracranial disorders, or systemic diseases such as metabolic-endocrinological conditions. It is difficult to establish the exact prevalence of cyclic vomiting syndrome because it is often misdiagnosed or unrecognized.

*Abdominal migraine* is an idiopathic condition, which is found mainly in subjects aged between 3 and 10 years of age, characterized by recurrent attacks of moderate to severe midline or poorly localized abdominal pain. The attacks are associated with vasomotor symptoms, nausea and vomiting, pallor and anorexia, lasting 2–72 h and there is a complete resolution between the episodes. Cephalalgic phase is typically not present during the attacks (28). The abdominal pain is often described as dull or just sore, not colicky, and interferes with daily activities in 72% of patients (46). The history and physical examination must exclude other medical conditions (gastrointestinal or urogenital diseases and central nervous system disorders). A careful history regarding headache must be taken and a diagnosis of migraine without aura should be considered if headache is present during attacks (28). Significant indicators for diagnosis are the absence of recurrent head pain, especially on initial presentation, and vomiting episodes that are less severe compared to cyclical vomiting syndrome (46). The abdominal pain resolves in 61% of patients, but 70% develop migraine with or without aura (47). Migraine therapies have been reported effective in treating abdominal migraine (48).

*Benign paroxysmal infantile vertigo* (BPV) is a disorder characterized by brief recurrent attacks of vertigo in otherwise healthy children. The vertigo is maximum at the onset of the attack and resolves spontaneously after a few minutes or a few hours without loss of consciousness. Nystagmus, ataxia, pallor, and vomiting may be associated symptoms (44). Between the attacks neurological examination, audiometric examination, and vestibular tests are normal. Posterior fossa tumors, epilepsy and vestibular disorders must be excluded (28). BPV has a prevalence of 2.6% in children. Onset is typically between 1 and 5 years of age and it is generally self-limiting within 10–12 years (49). BPV is the most frequent cause of vertigo in children aged 2 to 6 years and has a prevalence of about 2.6% in children from 5 to 15 years of age. In particular BPV account for 6.3% of the children who come to the emergency room for vertigo (50).

*Benign paroxysmal torticollis* (BPT) is a disorder characterized by recurrent episodes of head tilt to one side, with or without slight rotation, which resolve spontaneously after a few minutes to 30 days (28). The child's head can be positioned neutrally during attacks, although it is possible to find resistance during movement. This condition occurs in infants and young children with onset in the first year of life (44). Nausea, irritability, vomiting, pallor, drowsiness, eye abnormalities, dystonia, and nystagmus may be associated. Ataxia is seen more frequently associated in older children (28). Attacks occur from once a week to once every 5 months (44). Neurological examination is normal between the attacks. Differential diagnosis includes gastro-esophageal reflux, idiopathic torsion dystonia, and complex partial seizures, but special concern goes to the posterior fossa and cranio-cervical junction where congenital or acquired lesions may determine stiff neck (28). BPT management is essentially reassurance and supportive care (44).

### Tension-Type Headache

Tension-type headaches (TTH) are common in children with a prevalence of 5–25% in children and adolescents and an average onset age of ~7 years (26). The pain usually arises in the afternoon hours while the child attends school and the child often continues to practice his favorite activities despite severe or constant headaches (2). The average frequency of the attacks is about two per month and the duration about 2 h per single episode. In children TTH can be triggered by psychosocial stressors and anxiety; comorbidities frequently present are mood disorders, prevailing if the headache pain is chronic. It is not rare for the characteristics of tension headaches to change from pre-school age to adolescence. For this reason the so-called red flags must prompt further investigations on the tension-type headache. In particular, neuroimaging must always be sought in order to exclude life-threatening headaches. Often the symptoms of TTH can overlap with those of migraine and a migraine may transform over time to a tension type of episodic headache. Unlike migraine, TTH is not associated with photophobia, phonophobia or nausea, nor aggravated by physical activity. Furthermore, the pain is generally mild or moderate and not pulsating (28).

### Trigeminal Autonomic Cephalalgias

Trigeminal autonomic cephalalgias (TACs) are represented by various forms of headache syndromes such as: cluster headache (CH), paroxysmal hemicranias (PH), short-lasting unilateral neuralgiform headache attacks with conjunctival tearing and injection (SUNCT), and short-lasting unilateral headache attacks with cranial autonomic symptoms (SUNA) (28). The hallmark of these headache syndromes is the presence of unilateral, autonomic manifestations during the headache episode (3).

CH is a rare condition in children (from 0.03 to 0.1% of pediatric headaches) with a male preponderance (2.5:1). Only 5–10% of CH develop in childhood with a peak of onset in adolescence (mean age 11–14 years). A familiarity for CH is present in about 10% of pediatric cases compared to 25% for migraine (3). The clinical characteristics of pediatric onset CH, like in adults, are severe typically unilateral attacks of pain which are orbital, temporal, or in contiguous areas, lasting 15–180 min and which occur from one to eight times a day. Pain is associated with ispsilateral conjunctival injection on the same side as headache, watery eyes, nasal congestion, rhinorrhea, sweating, miosis, ptosis and/or palpebral oedema, and agitation (28). Tearing of the eye homolateral to the cephalalgia is the more frequent symptom of pediatric CH, followed by conjunctival injection and nasal secretion (3).

TACs share the clinical hallmarks of unilateral headache with prominent, ipsilateral, cranial parasympathetic autonomic features.

### Recurrent Painful Ophthalmoplegic Neuropathy

The previous and inappropriate nomenclature of this condition was “ophthalmoplegic migraine.” This terminology has been rejected, as this condition is not migraine, but rather a recurrent and painful neuropathy. It is a rare condition that occurs in 0.7/million people, in which headache is associated with partial or complete, unilateral paralysis of the oculomotor nerves. Clinically, it presents with repeated attacks of headache that have the characteristics of migraine, but are associated with loss of function of one or more oculomotor nerves (in most cases paresis of the third cranial nerve), in the absence of demonstrable brain lesions on neuroimaging (28). When the third cranial nerve is involved, the pupil is rarely spared, unlike what happens in the case of ischemic paralysis. This condition is found more in children than in adults and should be considered in the differential diagnosis of third nerve palsy in pediatrics (34). However, this disorder is rare and onset usually occurs before the age of 10. Injuries of the parasellar region or the upper orbital fissure and aneurysms of intracranial vessels should be excluded with appropriate neuroimaging studies. In some cases, MRI shows a capillation of gadolinium in the intracisternal portion of the affected cranial nerve, suggesting a recurrent demyelinating neuropathy (3), which may sometimes determine permanent cranial nerve deficit (51).

### Particular Forms “Sub Judice” Classified at Present in Appendix Section in ICHD-3 Revised

Infantile colic, alternating hemiplegic migraine, and vestibular migraine represent three additional forms that may be listed among “Episodic Syndromes” possibly associated with migraine (section 1.6 point in ICHD-3 revised) (28). Nevertheless, these conditions do not present sufficient evidence to be classified in this group (1.6) and require further studies.

*Infantile colic* affects one infant out of five and is defined as an episode of irritability, agitation, or inconsolable crying without a specific, apparent cause in an infant without stunted growth. Generally, the episodes last three or more hours a day for at least 3 days a week. Given the recent evidence of a possible association between migraine and infantile colic, the latter is now part of the “Episodic syndromes that can be associated with migraine” included in the appendix section of ICHD-3 (28).

*Alternating hemiplegia of infancy* (AHC) (or alternating hemiplegia of childhood) is a rare neurodevelopmental disease characterized by recurrent episodes of hemiplegia and paroxysmal disorders associated with persistent developmental delay and mental retardation. The incidence is about 1/100.000 newborns. AHC is classified by the ICHD-3 as an episodic syndrome that may be associated with migraine. The main features are recurrent episodes of intermittent hemiplegia, often migratory, and alternating associated with other neurological features such as dystonia, choreoathetosis, and developmental delay (44), lasting from a few minutes to a few days, with unilateral or bilateral onset. These episodic symptoms disappear immediately with sleep, but reappear after waking up with longer attacks. The diagnosis is primarily clinical and the initial signs are hemiplegia and dystonia in the first six months of life. Paroxysmal movements of the ocular globes appear in the first three months. An exclusion diagnosis is made on the basis of absence of epileptiform changes on EEG during the episodes.

Migraine-related syndromes [such as BPV and *vestibular migraine* (VM)] are the most common cause of episodic vertigo in children (52). In 35–60% of cases are associated with headache that can precede, follow or occur simultaneously with vestibular symptoms. The diagnostic criteria for VM proposed by Neuhauser (53), initially based on clinical and epidemiological observations of adult patients, have recently been validated (28). The clinician should arrive early to a reasonable diagnosis to start treatment early. This approach also minimizes parents' and children's anxiety, reduces interruption of leisure time and school activities, and prevents the development of VM.

Two other headache conditions are well-known to pediatricians, also in the emergency setting, as possible “variant forms of migraine”: Alice in Wonderland Syndrome and acute confusional migraine.

The *Alice in Wonderland Syndrome* (AWS) is a rare disorder first described by Todd and historically attributed to Lewis Carroll, author of the novel Alice in Wonderland. It seems that Carroll suffered from migraine and described in his famous story the symptoms that he himself presented. This syndrome of altered bodily perceptions consists of variations in dimensions and shape and distorted body images. Patients often narrate grotesque visual illusions, spatial distortions, micropsy, macropsy, metamorphopsia, and teleopsia (44). These experiences can precede or accompany a headache or occur without headaches. EBV infection appears to be the most common cause of AWS in children, unlike in adults where it occurs in conjunction with migraine episodes (54). It can also present in different disorders including epilepsy, drug intoxication, fever delirium, brain injury, schizophrenia, and hypnagogic states (44). Increasing scientific evidence of the relationship between migraine and AWS, means that in many patients it was considered an aura or equivalent migraine, particularly in children (54).

*Acute confusional migraine* (ACM) is a rare condition and the data reported is scarce. ACM is characterized by the onset of an acute confusional state which manifests itself in the form of agitation, memory impairment, disorientation, increased vigilance, dysarthria, or perceptive disorder. It occurs predominantly in late childhood and adolescence (50% of cases) and there is often a positive family history for migraine. The headache can appear before, during or after the confusional state, lasting a few minutes to a few hours. Resolution within 24 h and often associated with retrograde amnesia. Encephalitis, convulsions, strokes, vasculitis of the central nervous system, metabolic encephalopathy, toxic ingestion, and other causes of acute confusion must be excluded. During the disorder, the EEG can detect a generalized slowdown and sometimes an intermittent frontal rhythmic delta activity (44).

## Secondary Headaches

According to the ICHD-3, a new headache of recent onset that presents with another disorder recognized as capable of prompting it, is always diagnosed as secondary.

Secondary headaches in the pediatric population are more frequently due to non-LT diseases such as upper respiratory tract infections, sinusitis, and systemic infections. In a minority of patients, headache is secondary to serious LT intracranial disorders such as brain tumors, hydrocephalus, idiopathic intracranial hypertension, brain abscess/meningitis, aneurysm and vascular malformation, intoxication, and ventriculo-peritoneal shunt malfunction. LT causes of headache are found in patients whose clinical history and physical examination reveal so-called “red flags” (36, 55) (Table 6).

Thunderclap headache is a severe and acute headache that reaches its maximum peak intensity in about a minute and lasts about 5 min. This form of headache can be associated with a considerable number of potentially LT disorders (28) so it is essential to carry out imaging tests to exclude them.

Headaches due to increased intracranial pressure are associated with pain that wakes the patient at night and pain early in the morning (55). However, 25% of children with episodes of primary headache wake up at night. In these cases the pain usually starts before the child goes to sleep. The importance of a good and detailed history should always be stressed, together with a careful and oriented physical examination, fundamental in identifying these cases.

The role of the ED physician is to identify the causes of headache that require rapid intervention. Failure to do so may have devastating consequences for children.

### Thunderclap Headache

Thunderclap headache (TCH) is an acute onset headache that quickly reaches its maximum intensity level in a minute and lasts about 5 min (56). This type of headache in adults is described as “the worst headache ever had.” In children, it represents a medical urgency since this form of acute onset headache is mainly associated with LT causes for which rapid diagnosis and prompt treatment are essential (57).

Various conditions can be associated with TCH including: leaking intracranial aneurysm; cervical arterial dissection; venous sinus thrombosis; reversible cerebral vasoconstriction syndrome; pituitary apoplexy; posterior reversible encephalopathy; hypertensive crisis; spontaneous intracranial hypotension. Very rarely, TCH may represent a primary form but a diagnosis can be made only after other etiologies have been excluded by appropriate investigations such as computed tomography (CT) angiography (CTA), magnetic resonance imaging (MRI), magnetic resonance angiography (MRA), magnetic resonance venography, and cerebrospinal fluid evaluation (38).

*Cervical artery dissection* in children occurs with a TCH in 25% of cases (58). Laceration of the vascular wall determines extravasation of blood within the vessel wall which occludes the distal portion of the vessel involved. For this reason, classic dissection of carotid or vertebral vessels in children presents with acute onset headache associated with neck pain and/or tenderness and ipsilateral supraorbital, auricular, or mandibular pain (57). Focal neurological deficits may be other presenting symptoms. In these cases it is therefore mandatory to perform diagnostic imaging and proceed with the appropriate treatment.

*Venous sinus thrombosis* begins with headache in more than 75% of cases in childhood (59). The headache is mainly severe with a chronic, progressive pattern that worsens over days or weeks. However, about 10% of children present an “explosive headache” onset triggered by coughing, sneezing and/or change in position (57). Other symptoms associated are vomiting, diplopia and papilledema, and convulsions. Dehydration is a predisposing factor in patients with infectious conditions or pre-existing brain lesions (58). For the diagnosis it is mandatory to perform a CT scan with contrast medium in an emergency setting though the gold standard remains MRI if available in the ED.

The *reversible cerebral vasoconstriction syndrome* (RCVS) is a clinical condition of transient dysregulation of the cerebral vascular tone. An acute and transient malfunction in control of the intracranial vascular tone causes narrowing and micro-dilatation of a cerebral area, characterized by a mainly favorable outcome (60, 61). RCSV may be primitive or a complication of other clinical conditions such as cerebral infarctions, intracranial hemorrhages or cerebral edema. The classic clinical manifestation is the sudden onset of recurrent headaches that are brief, but extremely painful and associated with an altered level of consciousness and/or focal neurological symptoms. In these case the physician must use diagnostic imaging (MRI, angiography) (57). RCVS treatment is the elimination of possible triggering factors, together with symptomatic measures.

Spontaneous *intracranial hemorrhage (ICH) and ischemic stroke* are rare causes of headache in children. Although an acute TCH is the classic presenting symptom of ICH, most children who have ICH or ischemic stroke have additional signs or symptoms by the time they present to a medical facility. ICH should be considered in patients who have an acute onset of severe headache, particularly if the patient has an abnormal neurologic examination or a disorder that places him or her at risk for hemorrhage (62).

### Infection

Infections of the central nervous system must always be suspected in case of a patient with headache, systemic symptoms (in particular fever) and altered consciousness (38, 62). Photophobia and neck stiffness are frequent symptoms in children with meningitis. Hypotension or the presence of hemorrhagic skin lesions (petechiae and/or ecchymosis) are also considered red flags for central nervous system infections and warrant immediate lumbar puncture with cerebrospinal fluid examination (60). In children with fever, localized headache and focal neurological deficits, a brain abscess should be considered especially if there is a recent history of otitis media, mastoiditis, endocarditis or immunosuppression (57). In encephalitis (viral, bacterial, and/or autoimmune) the distinctive sign is headache in association with a variety of psychiatric and behavioral symptoms such as hallucinations and psychosis, convulsions, memory dysfunction with short-term memory loss, language disorders and altered level of consciousness (2).

### Intracranial Masses

Brain tumors are rare in children with an incidence of 5 per 100,000 between 0 and 19 years of age (2), and delayed diagnosis can affect negatively morbidity and mortality. The characteristics of headaches due to brain tumors are generally linked to the position, size, and rate of growth of the mass. Prevalence of brain tumor in pediatric patients accessing the ED varies from 0.4 to 3% in relation to the studies examined (Table 1). In most cases it is a chronic and progressive headache with frequent nocturnal awakenings or a morning headache with or without vomiting. Exacerbation of the headache with Valsalva, cough, and change of position occurs in a minority of patients. Patients are often unable to give a specific location of the pain. However, supratentorial tumors affecting the structures innervated by the ophthalmic branch of the trigeminal nerve can sometimes produce a frontotemporal headache, whereas posterior fossa tumors that compress the glossopharyngeal and vagus nerve may cause occipital-nuchal pain (2). In a study on 393 pediatric patients diagnosed with a brain tumor in the ED, emerged that the posterior fossa was the site of tumor in 48.3% of cases, supratentorial in 21.8%, brainstem in 16.1%, and central in 13.8% (63). This same study underlined that the mean onset of any symptom was 86.3 days and for headaches was 104.5 days before the diagnosis was made in the ED. Symptoms or signs reported were headache (66.7%), hydrocephalus (58.6%), nausea/vomiting (49.4%), gait disturbance (42.5%), vision problems (20.7%), seizure (17.2%), change in behavior/academic performance (17.2%), cranial nerve deficits (16.1%), altered mental status (16.1%), back/neck pain (16.1%), papilledema (12.6%), facial asymmetry (10.3%), sensory deficits (8%), focal motor weakness (6.9%), cranial nerve deficit (6.9%), ptosis (5.7%), macrocephaly (4.6%), asymptomatic (3.4%), and anisocoria (1.1%) (63). Headache and vomiting are the most common and early symptoms in children with brain tumors. However, symptoms or visual signs and behavioral changes are often present. Abnormalities in neurological examination are reported in most of the children. Symptoms of intracranial hypertension suggest the need for a neurological clinical examination and an ophthalmological assessment. Among children and young adults with an intracranial tumor, non-localizing features such as headache, vomiting, lethargy, drowsiness, failure to thrive, parental concern, and features of raised ICP were far more common than specific features, such as focal neurological deficits, prior to diagnosis. In all age groups, cranial nerve II, III, IV, or VI dysfunction was also common. Many of these symptoms occurred with increasing frequency with tumor progression. Signs of raised ICP become the most common group of presenting features in the final month before diagnosis (64, 65).

### Idiopathic Intracranial Hypertension

Idiopathic intracranial hypertension (IIH) or pseudotumor cerebri is a condition characterized by increased intracranial pressure (>28 cmH_2_O) in the absence of clinical or radiological evidence of an intracranial lesion recognized as capable of causing it. In these patients ophthalmic assessment shows bilateral papilledema while neuroimaging is found to be within normal limits. Neuroimaging findings more easily associated with the diagnosis of IIH include empty turcic saddle, distention of the perioptic subarachnoid space, compression of the posterior sclerae, cupping of optic disks and distension of the optic nerve sheaths, and transverse cerebral venous sinus stenosis. Normal physical chemical examination of cerebrospinal fluid (CSF) with high opening pressure confirms the diagnosis of IIH (28, 66). For diagnostic purposes, CSF should be measured in the absence of treatment to lower intracranial pressure. IIH occurs in obese adolescent females (28). Visual acuity loss is reported in 6–22% of children and visual field loss occurs in up to 91% at clinical onset. In our experience it is useful to evaluate serum electrolytes in patients with suspected IIH as this condition can be associated with underlying hypocalcemia. The role of neuroimaging and ultrasound-based optic nerve sheath diameter measurement has significantly changed the evaluation of IHH patients.

## Evaluation of Headache in Ed: *How to Diagnose Headache*

### History

History and physical examination are extremely important in the evaluation of children who present headaches. They are reliable indicators of headaches secondary to life-treathening conditions. An accurate history is crucial for a correct diagnosis, so it is important to ask the right questions to both the child and the parents. The emergency physician should ask about headache description (onset, duration, quality, and pain severity), triggers and exacerbating factors (stress, sleep pattern changes), alleviating factors (Table 7), and specifically search for warning signs (Table 6). Background information must be investigated, such as drug use, systemic disease (sickle cell disease, immunodeficiency, malignancy, pregnancy, neurocutaneous syndrome, or congenital heart disease), associated symptoms, trauma, and family history.

**Table 7:** Key questions in taking the clinical history in a child with headache.

Acute headache	Tinte of onset Duration Localization Quality Intensity Premonitory symptoms Aura Associated vegetative symptoms Impairment of daily routine Ameliorating factors Aggravating factors Triggering factors Factors possibly associated to onset Efficacy of medications taken
Additional features inrecurrent headache	Number of headache types Frequency Sequence of typical episode Impairment of quality of life

*Modified by Ozge et al. (1) and Papetti et al. (35)*.

A systematic review (36) proposed to distinguish between “high risk red flags” and “relatively red flags” (Table 6) which are outlined in detail in this review. Any high risk red flag should suggest performing neuroimaging. In the case of relatively red flags, a more restrained approach can be appropriate on the individual setting (36). Household and family dynamics, psychosocial stress factors, and school performance should also be evaluated because they can be precipitating factors in children and adolescents. A HEADSS (home, education, alcohol, drugs, smoking, sex) screen should be performed in all adolescent patients (16).

Lewis et al. (55) suggested classifying headaches into four temporal patterns (acute, recurrent acute, chronic progressive, chronic non-progressive; Table 8). Acute headaches which evolve suddenly can be considered suggestive of organic disease and must be evaluated very carefully. Fever during upper respiratory tract infection and sinusitis are the most frequent causes of sudden onset of pediatric headache (1). The acute-recurrent pattern of episodic headache, separated by symptom-free intervals, occurs in migraine, tension-type headache, cluster headache, neuralgias, and epileptic variants (55). Migraine episodes occur more frequently as acute recurrent headaches with chronic pain episodes. If ICHD-3 criteria for a primary headache disorder are met, no further investigations are necessary (36). Chronic progressive headaches are particularly concerning because they are generally associated with conditions characterized by gradual increase in intracranial pressure (brain tumor, hydrocephalus, IIH, brain abscess, aneurysm, and vascular malformations, intoxication) (1, 36). The chronic non-progressive and mixed patterns usually fall within the spectrum of chronic daily headache with or without superimposed migraine or analgesic abuse (55). Headache on awakening or sleep interruption due to headache have been commonly regarded as a potential sign of raised intracranial pressure and, therefore, significant underlying pathology, but this issue is debated and warrants further investigation. Medina et al. (67) found persistent headache that wakes a child repeatedly from sleep or occurs immediately on awakening, with no family history of migraine, to be the strongest predictor of intracranial lesions that require neuroimaging. Nevertheless, a recent study of Ahmed et al. (68) reported that neuroimaging among 4% of patients with headache sleep interruption or on awakening revealed intracranial abnormalities that were unlikely to have caused the awakening and none of them required prompt intervention. The authors concluded that awakening or sleep interruption due to headache among clinically well and neurologically normal pediatric patients was most likely to be caused by primary headaches, particularly migraine or tension type headaches, and this needs to be more widely recognized in order to avoid unnecessary brain imaging.

**Table 8:** Causes of headache by temporal pattern.

**Acute headache**	**Acute recurrent headache**
Upper respiratory tract infection, with or without fever Acute sinusitis Pharyngitis Meningitis (viral or bacterial) Migraine (first attack) Post-ictal headache Hypertension Substance abuse (e.g., cocaine) Medication (e.g., methylphenidate, oral contraceptives, steroids) Intoxicants (e.g., lead, carbon monoxide) Ventriculoperitioneal shunt malfunction Brain tumor Hydrocephalus Subarachnoid hemorrhage Intracranial hemorrhage Venous sinus thrombosis	Migraine Tension-type headache Cluster headache Seizures Hypertension Hyperthyroidism Pheochromocytoma Medication-induced headache MELAS
**Chronic progressive headache**	**Chronic non-progressive headache**
Brain tumor Hydrocephalus (obstructive or communicating) Pseudotumor cerebri Brain abscess Hematoma (chronic subdural hematoma) Aneurysm and vascular malformations Medications (e.g., birth control pills, tetracycline, vitamin A) Intoxication (lead poisoning)	Chronic migraine Chronic tension-type headaches(analgesic overuse) Post-concussive syndrome Temporomandibular joint syndrome Cluster headache

*Adapted by Lewis et al. (55) and Papetti et al. (35)*.

### Physical Examination

The general physical examination is of extreme importance and must be conducted in a complete manner. The first step is to assess the patient's severity of pain, which may be indicative of a more serious underlying condition (14, 16), further to investigate the clinical features of children presenting to a pediatric ED with headache as the chief complaint and report in their observational study that all patients with LT secondary headache can present very intense pain. One must investigate for important clues leading to the correct diagnosis (skin rash, petechiae, stiff neck, organomegaly). Altered vital signs (in particular body temperature and blood pressure) are suspect for serious conditions (69). When evaluating of a child with headache it is mandatory to perform a complete neurological examination aimed at identifying signs of intracranial lesion. Particular attention must be paid to the level of consciousness, meningeal signs, visual disturbances, focal neurological deficits, disorders of gait and coordination, speech and hearing disorders, and localized altered sensitivity of the scalp or any area of the body. In younger patients, it may be useful to evaluate the head circumference. The head and neck should be inspected and palpated, investigating for visual signs (i.e., unequal pupils) and sinus tenderness. The skin should be searched for possible signs of a neurocutaneous syndrome, in particular neurofibromatosis and tuberous sclerosis, which could be indicative of intracranial neoplasms (70). In addition, a psychiatric evaluation of children and parents should be performed when needed. In the majority of patients with primary headache disorders, general physical and neurological examination are both normal (1).

Red flags (Table 6) at physical examination should include headache with signs of systemic disorders (skin rash, petechiae, stiff neck, organomegaly), focal neurological signs, symptoms of disease (other than typical aura), and papilledema (35, 36).

Occipital headache is considered a risk factor for serious secondary headache, but it is currently under debate whether to consider it an absolute or relative red flag. Some authors (14, 55) aimed to identify clinical clues for headaches associated with serious LT intracranial disorders. They agreed that occipital location and the inability of the child to describe the quality of his pain are risk factors that require further investigation. In contrast, Genizi et al. (33) in a retrospective study of 314 pediatric patients with headache (39 patients with occipital headache), reported that etiology of occipital headaches does not differ from other sites, suggesting that occipital headaches should not be evaluated differently from other headaches.

### Diagnosting Testing

The few children that need further evaluation should have the work-up guided by the underlying cause suspected. Diagnostic tests are varied; they include routine laboratory analysis, CSF examination, and neuroimaging with CT or MRI. Routine neuroimaging is not indicated and guidelines recommend that it be performed in children presenting with an abnormal neurological examination and a history of CNS disease (71).

#### Fundoscopic Examination

Ophthalmologist consultation is frequently requested by the emergency room physician to rule out papilledema (optic disc swelling) in patients with headache (72). Papilledema can be a sign of increased intracranial pressure and is believed to develop from hours to weeks after the onset of the headache. A meta-analysis of about 400 pediatric patients with brain tumors reported the presence of papilledema in only 13% of patients (73). Segev-Becker et al. (74) analyzed 479 children with headache in the ED to investigate for papilledema. Only six children (3.5%) had papilledema (four IIH, one meningococcal meningitis, and one patient was lost to follow-up). Furthermore, medulloblastoma was diagnosed in one of the patients with normal funduscopic examination. The authors point out that it is not useful to evaluate routinely the ocular fundus in the emergency room, especially if the onset of symptoms is <24 h (74). However, we believe that fundus examination should always be included in the neurological evaluation of a child with headache even though it does not always exclude dangerous secondary conditions.

#### Neuroimaging

Assessment and diagnosis of headaches can be very difficult for pediatricians and neuroimaging (CT or MRI) is often required as part of the investigations (70). CT is the first neuroimaging performed in the ED in patients with suspect secondary headache because it is a fast and easily performed test. Modern CT machines used in pediatrics have developed low-dose radiation systems therefore representing the first-instance examination in an emergency setting. MRI provides superior quality images but is more expensive and children under 6 years of age may need sedation or anesthesia to perform it (24, 70).

Studies on the utilization of neuroimaging in the ED show that the frequency of pathological findings that lead to significant variation in management are rare, about 1.2% of neurologically normal patients (75). Neuroimaging techniques should be reserved for children with a suspicious clinical history, abnormal findings on neurological examination or other symptoms suggestive of intracranial space-occupying lesions (76). Therefore, before deciding on neuroimaging, it is essential to have complete information regarding age at onset of headache, type of onset (abrupt or gradual), frequency, severity, presence of an aura, as well as perform a thorough clinical and neurological assessment. In ED, the headache characterization is mandatory in order to identify patients who would benefit from neuroimaging. At the same time it is essential to identify children who can do without neuroradiological investigations to avoid subjecting them to useless and potentially harmful procedures (70). A headache that appears during or after treatment of otitis media or sinusitis may indicate the possible intracranial spread of the infection. In the literature intracranial complications are reported in about 3% of patients with sinusitis being represented by epidural and subdural empyema, followed by brain abscess (77). In the subdural empyema the patient is usually febrile with associated neurological symptoms such as altered state of consciousness, focal neurological deficits, and signs of meningeal irritation (77, 78). Sinus thrombosis is a possible complication of otitis media and mastoiditis (77). In infants and toddler, signs of intracranial mass include the presence of an increase in head circumference, prominent scalp veins, and disjunction of cranial sutures. In older children the most frequent disorders are represented by headache or diplopia. Signs of brainstem herniation are bradycardia, arterial hypertension, and abnormal in inspirations; these features should require prompt urgent neuroimaging (23). Red flags are the basis of existing guidelines and recommendations regarding the use of neuroimaging (Table 6). However, there is no clear consensus on which findings should be used for decision making, resulting in a large number of findings proposed to be concerning enough to warrant urgent imaging. Tzse et al. (76) enrolled 224 patients of which 197 (87.9%) had at least one red flag in their history, including headache waking from sleep (34.8%), headache present upon or soon after waking (39.7%), or headaches increasing in frequency, duration, and severity (40, 33.1, and 46.3%). The prevalence of urgent intracranial abnormalities was 1%. Abnormal neurological exam, extreme pain intensity of presenting headache, severe vomiting especially early in the morning, and positional symptoms were independently associated with emergency neuroimaging. These data suggest that many children with headache receive unnecessary neuroimaging due to the high prevalence of non-specific red flag findings (76). The yield of neuroimaging in pediatric headache with normal neurological examination is low. However, in patients with positive neurological signs or symptoms the likelihood of positive neuroimaging findings is high (79). In fact, the presence of ataxia, focal crises, dysfunction of the cranial nerves, nystagmus, and abnormal reflexes are indications to perform neuroimaging. Symptoms of increased intracranial pressure such as papilledema, increased head circumference (in the youngest), vomiting, mood or behavior changes, and altered mental status are further indications.

We must keep in mind that children under age 5 years still represent a further challenge in the emergency setting for the pediatrician or neurologist. In this age group it would seem that headache may be the only symptom. Nevertheless, a study examining 364 children (between 2 and 5 years of age) with headache demonstrated that diagnostic yield of CT scans is low for children who present no worrying history and a normal neurological examination (23). A previous analysis of data regarding more than 3,000 children with brain tumors showed that 98% had one of the following five signs: papilledema, ataxia, hemiparesis, abnormal eye movements, or depressed reflexes (80). In the case of abnormal neurological examination or suspicious clinical history, imaging should be performed. MRI is preferable, but CT without contrast agent, because of its accessibility and rapidity, is acceptable as a routine protocol in ED (23). Proper neuroimaging of children with headache is very specific to the headache type. The choice of the most correct sequences, CTA or if we use MRI (i.e., MRA, T2-weighted gradient-echo, diffusion-weighted sequences, and post-gadolinium-enhanced sequences), is fundamental to perform the most appropriate path to arrive at an etiological diagnosis by maximizing the capacity of the imaging technique with the minimum risk of the child [see ACR Appropriateness Criteria Headache Child for more detailed information ref. (81)]. In emergency setting some centers prefer the use of CTA over MRA so we would suggest considering both (one or the other) when vascular imaging is indicated.

#### Laboratory Tests, Lumbar Puncture, EEG, and Neurophysiological Examinations

Laboratory tests are rarely useful in the evaluation of headaches (69). They can be useful just to demonstrate that the most frequent type of secondary headache in children admitted to the ED is related to upper respiratory tract infections. Lumbar puncture (LP) is not routinely recommended in the assessment of headaches in children. This procedure should be performed in children with suspected intracranial infection, subarachnoid hemorrhage or IIH (72).

EEG and neurophysiological examinations (including evoked cortical potentials) are not routinely used in the diagnosis of children with headache in the ED (71). In particular, EEG is not necessary for distinguishing a primary headache disorder in children from secondary headache due to head and neck structural disease, or those due to a psychogenic cause (55). Its' use is limited to “migraine-triggered seizures” (migralepsy concept) (20, 82) and to the rare cases of “Ictal Epileptic Headache” [see the criteria by Parisi et al. (83)]. Recently, the “migralepsy concept” has been seriously questioned, in favor of the concept of “Hemicrania Epileptica,” which is an ictal epileptic headache followed and/or associated with other motor, sensory, and autonomic signs/symptoms (84).

## Treatment

Appropriate advice and treatment requires consideration of a wide differential diagnosis between primary and secondary headaches, as well as the different types of primary headache. In the ED general measures include stabilization of the airway, breathing, and circulation in critical patients. In patients in good general condition, the treatment should include placing the child in a quiet, dark room where he can rest since sleep is often the most effective treatment. Diagnosis and therapeutic decisions are often complicated by comorbidities, and different primary headaches can co-exist. Being familiar with general pediatrics, and pediatric headache disorders in particular allows for better advice and better treatment options for the patients (85). The three major domains of headache treatment in a pediatric ED include lifestyle changes, abortive therapy and complementary therapies (62).

### Non-drug Treatments and Advice

Children will often naturally seek out dark, quiet area when they have a headache and this should be encouraged. They should also be encouraged to take frequent, small sips of water to remain hydrated. If they are in a place where they can fall asleep, sleep may be useful in terminating a migraine attack.

A variety of physical, complementary, and lifestyle interventions are available and summarized by the SMART acronym (get sufficient and appropriate Sleep; regular healthy Meals; appropriate Activity neither excessive nor deficient; consider methods of Relaxion; recognize, and avoid Trigger); though mostly with poor empirical evidence of efficacy (85). Excess caffeine, aspartame, monosodium glutamate, nitrite, alcohol, and chocolate can cause headaches. Therefore, we advise not to exceed with these substances though the role of exclusion diets in infantile migraine is still not demonstrated.

### Acute Treatment

Treatments for migraine include symptom relief of acute attacks (Table 9). Usually the therapies aim to eliminate head pain and reduce the associated symptoms, such as nausea, phonophobia, and photophobia. Since acute medications are most effective when taken while pain is still mild, which tends to be early in an attack, families and adolescents should work out strategies to ensure that the medications are available and on hand (86). During acute head pain or exacerbation of chronic headache, nausea, and/or vomiting may make oral administration of medications difficult (85). Vomiting can sometimes be a very important part of a migraine attack in young children. In these cases, it is a priority to start intravenous (IV) rehydration and to administer antiemetic drugs (Table 10). When it is impossible to administer drugs by mouth, IV administration should be considered in the pediatric ED.

**Table 9:** Abortive therapies for pediatric migraine.

**Drug**	**Usual Dosage**
Ibuprofen	10 mg/kg every 6–8 h Age > 12 y to adult: 400–600 mg every 6 h Max: 2,400 mg/day
Naproxen sodium	5-7 mg,kg every 8–12 h Age > 13 y to adult: 250–500 mg every 8 h Max: 1,250 mg/day
Acetaminophen	10–15 mg/kg every 4–6 h Age > 13y to adult: 650–1,000 mg every 6-8 h Max: 3,000 mg/day
Rizatriptan	Children < 40 kg: 5 mg PO once Children > 40 Kg: 10 mg PO once Max: 30 mg/day (propranolol will increase serum concentration of rizatriptan)
Zolmitriptan	Nasal Children > 12 y: 2.5–5 mg IN once Max: 10 mg/day Oral (tablet or ODT) Max: 10 mg/day
Sumatriptan	Nasal Age 4–6 y: 5 mg Age7–11 y: 10 mg Age > l2 y:20 mg Subcutaneous Child: 0.06 mg/kg, age > 12 y: 6 mg Oral Child: 1 mg/kg, max 50 mg/day
Almotriptan	Age > 12 y:6.25–12.5 mg PO, may repeat once in 2 h Max: 25 mg/day
Sumatriptan/naproxen	Age 12–17 y: 1 tablet 10 mg sumatriptan/60 mg naproxen, max dose 85 mg sumatriptan/500 mg naproxen

*IN, Intranasal; Max, Maximum; ODT, Orally disintegrating tablet; PO, oral*.

**Table 10:** Antinausea/vomiting medication options in pediatric migraine.

**Drug**	**Dose**	**Toxicity**
Prochlorperazine	Oral Child: 10–13 kg: 2.5 mg every 12–24 h Child: 13–18 kg: 2.5 mg every 8–12 h, max 10 mg/day Child: 18–40 kg: 2.5 mg every 8 h, or 5 mg every 12 h Intravenous Child: 0.1–0.15 mg/kg/dose	Sedation Dystonic reaction
Promethazine	Oral or Rectal Child: 0.25–1 mg every 4–6 h	Sedation Dystonic reaction
Ondansetron	Oral 4–8 mg every 8 h <15 kg: 0.2 mg/Kg 15–30 kg: 4 mg >30 kg: 4–8 mg	Sedation Dystonic reaction

Oral analgesics such as paracetamol (10–15 mg per kg) and ibuprofen (10 mg per kg) are the mainstay of acute therapy for headache in pediatrics (6). Often the child or adolescent has already taken analgesics without apparent benefit, so one should check that the drug has been taken with the correct dosage and give indications for repeat administration.

Other drugs such as ergot derivatives (e.g., dihydroergotamine) and triptans (serotonin 1b/1d receptor agonists) have demonstrated efficacy in adults. Many of these medications have now been studied in children and adolescents and some have been approved for use in the pediatric age group. Four kinds of triptan are labeled by the US Food and Drug Administration for acute migraine in adolescents 12–17 years of age: almotriptan (oral), zolmitriptan (nasal spray), rizatriptan (oral), and sumatriptan/naproxen (oral); and one medication, rizatriptan (melt), is labeled for use in children 6 years and older.

A recent Cochrane (87) examined 27 randomized controlled pediatric trials of drugs compared to placebo to assess the efficacy in providing pain relief 2 h after acute headache treatment. Based on a systematic review, ibuprofen seems more effective, making it an excellent choice for the treatment of head pain; paracetamol has not been shown to be effective in providing headache relief. In a small cross-over study, predominantly in children, oral paracetamol was not superior to placebo or ibuprofen (88). Triptans were more effective than placebo in determining pain relief in 3 studies involving children and 21 studies involving adolescents and no significant difference was observed between the subgroups of triptans (including almotriptan, eletriptan, naratriptan, rizatriptan, sumatriptan, and zolmitriptan). No difference was found between oral and intranasal administration of triptans in terms of efficacy, though the oral form is better tolerated (86). No patient in the studies analyzed reported serious side effects after administration of triptans. Side effects, usually mild, included fatigue, dizziness, asthenia, dry mouth, and nausea or vomiting with oral preparations, and taste disturbance, nasal symptoms, and nausea with intranasal preparations. The combination of sumatriptan with naproxen sodium was also more effective than placebo in producing pain relief in a study in adolescents (86).

Children with long-lasting migraine or status migrainosus may need ED treatment, if home therapy has failed and symptoms remain debilitating.

*Dopamine receptor antagonists* (chlorpromazine, prochlorperazine, promethazine, metoclopramide) are possible therapeutic options in the acute treatment of pediatric headache for their ability to control both pain and nausea/vomiting, always considering that the possible side effects include appearance of extrapyramidal reactions and sedation (89). Acute treatment of head pain via saline infusion, with intravenous ketorolac (0.5 mg/kg with a maximum dose of 30 mg) and prochlorperazine (0.15 mg/kg with a maximum dose of 10 mg), given intravenously, proved effective with a reduction of pain within 60 min in 55% of patients treated (90). A recent study comparing the effectiveness of three commonly used parenteral dopamine antagonists (prochlorperazine, metoclopramide, and promethazine) to abort pediatric migraine, found that promethazine was significantly associated with higher odds of treatment failure leading to opioid administration and poor pain control. The authors recommend using prochlorperazine or metoclopramide instead of promethazine for pediatric ED migraine treatment (91).

*Dihydroergotamine (DHE)* has shown efficacy and good tolerability in the treatment of pediatric migraine. The drug is usually administered in a hospital setting at both a high (0.5–1.0 mg/kg every 8 h) and a low dose (0.1–0.2 mg/kg every 8 h) (92), but there are still few studies in the pediatric population (93–95).

The usefulness of IV DHE in pediatric migraine has been suggested by two small retrospective studies of a total of 62 children and adolescents admitted to hospital for refractory migraine or status migrainosus (93, 94). Pain-free status upon discharge was reported in 74.4% of patients following repeat administration of DHE (0.1–0.5 mg per dose; on average 5–7 doses per patient). Nevertheless, these studies were weakened by prior treatment of the subjects with dopamine agonists. A recent study on treatment of 145 patients with pediatric refractory headache (only 28 with status migrainosus) showed that most responded to intravenous therapy with DHE, but complete resolution was more easily achieved in children with status migrainosus (96). However, only seven patients had headache duration of less than a week and it is likely that some cases reported as status migrainosus were actually worsened chronic migraine.

There is a lack of evidence regarding the use of *bolus IV fluids*, despite the fact that many protocols in the ED include this treatment. Only one single blinded randomized controlled trial is present in literature with patients divided into two groups: A (no medication given in combination with IV fluid 10 mL/Kg) and B (medication may be given simultaneously) (97). The authors conclude that the overall decrease in pain, measured with VAS scale, with IV fluid was small and no statistically clinical difference was found. Treatment with IV fluid hydration did not significantly influence headache relief at 30 min in children or adolescents with migraine in the ED. However, a clinically meaningful response was observed in 17.8% while recurrence of headache after ED discharge was 33%.

*Sodium valproate IV* could represent a treatment option for acute migraine, as suggested by two small retrospective studies on 31 and 12 adolescents (98, 99). Only the second study (99) was carried out in the emergency setting and pain reduction was 39.8% with time to maximum relief of 63 ± 31 min at a dose of 100 mg. Three adolescents required a second dose of 500 mg that was infused over 14 ± 6 min, determining a 57% reduction in pain intensity from baseline.

*Opioid medications* are explicitly discouraged for primary headache disorders because they may potentiate migraine pathophysiology at the molecular level by blunting the response to targeted abortive therapies, converting episodic to chronic headache (100, 101). In fact, the American Academy of Neurology published a statement recommending against the use of opioids for primary headache (102). The increased use of opioid analgesics to treat pain has been concurrent with the rising rate of prescription opioid abuse and related morbidity and mortality, especially in adolescents (103, 104). Nevertheless, opioids were prescribed for pediatric pain, including headache, particularly in the ED setting (103–106). However, in a very recent study it emerged that opioid prescribing rates for pediatric headache were low compared to adults, with a decreasing temporal trend. Nonetheless, ED prescribing rates were 4-fold higher than ambulatory care settings, though pediatricians, also in the ED, prescribed opioids less frequently. This was especially true for children seen in a pediatric hospital compared to a generalist hospital (107) or tertiary care vs. community based ED (106). Opioids are not part of the American Academy of Neurology practice parameter for pediatric pharmacological treatment of headache (71). At present they do not have a scientific evidence of efficacy in the pediatric population and they seem to be associated with an increase in hospitalization time (108).

#### Treating Medication Overuse Headache

Sporadically you can observe in pediatric EDs an acute attack in adolescents affected by medication overuse headache (MOH). These patients, who previously had episodic tension-type headache, migraine without aura or migraine with aura, develop a chronic headache(≥15 days a month for ≥3 months) while taking the following drugs, alone or in combination:

- triptans, ergot alkaloids, opiates or combination analgesics for ≥10 days a month;
- paracetamol, aspirin, or any non-steroidal anti-inflammatory drug for ≥15 days a month. MOH typically presents when a bad patch of migraine with or without aura has transformed into a chronic daily headache, including chronic migraine (85).

The suspect medication must be withdrawn abruptly and advice given on alternative treatments including, in some cases, prophylactic medication. Complete remission after withdrawal is no longer a diagnostic criteria (109).

## Conclusion

Most of the pediatric headaches in the ED are secondary benign headaches related to acute upper respiratory tract infections or a primary headache syndrome. Nevertheless, we must be able to detect the associated signs and symptoms of secondary headache; both the more frequent, non-LT conditions and the less frequent, severe conditions (i.e., brain tumors or other intracerebral space occupying lesions). A stepwise approach to pediatric headaches is essential to avoid missing secondary headaches and to promptly make the correct diagnosis. A complete history is paramount, including features of the headache and its characteristics, family and social history, and risk factors for systemic illness, as well as the symptoms or factors associated with the headache. A detailed physical and neurological examination, with attention to abnormalities that could be associated with a secondary cause of headache, is important for the subsequent diagnostic workup. Fundoscopic evaluation, in our opinion, should be part of the neurological examination of these patients and could be extremely useful in identifying doubtful cases. One must always search for red flags, distinguishing “high risk red flags” and “relatively red flags.” Any “high risk red flag” should prompt neuroimaging while, in the case of “relatively red flags,” a more restrained approach can be appropriate in the individual setting. Neuroimaging and other tests must be performed for positive findings on neurological evaluation or if there is concern for a secondary cause of headache in the history or physical examination. LP should be performed in case of suspected meningitis and it is diagnostic in the case of IIH. If ICHD-3 criteria for primary headache are not fulfilled, further investigations may be necessary. In patients with headache it is essential to treat the acute episode immediately according to evidence based medicine. When migraine is suspected, the administration of NSAIDs and triptans should be considered. In case of nausea and vomiting, antiemetic drugs and IV rehydration should be administered. Indications on lifestyle changes and a diary of headaches are useful in these patients. New symptoms and reactions to treatment should prompt review of the initial plan and appropriate changes.

**Figure 1:**
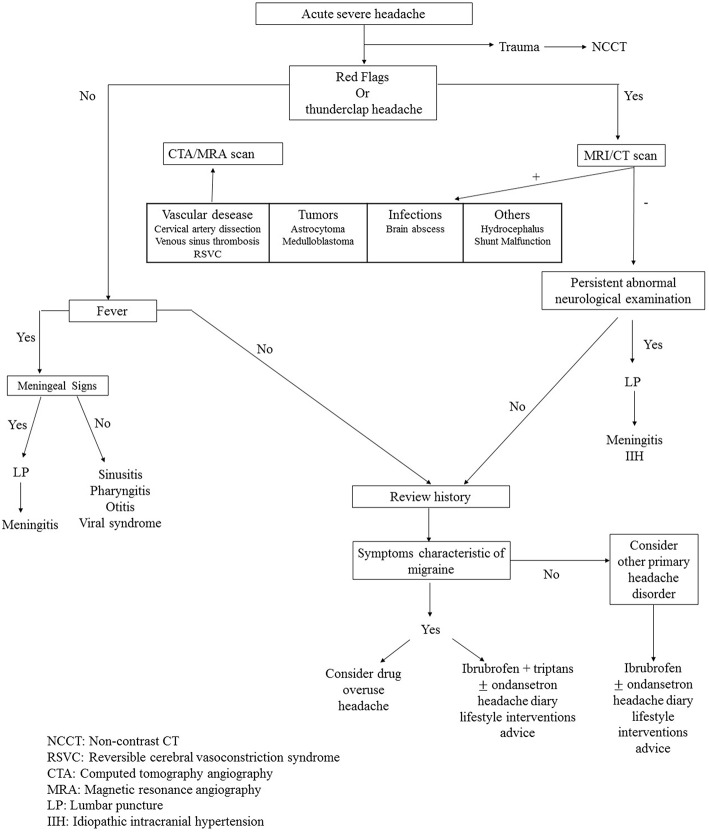
Clinical pathway of pediatric patient with headache in emergency setting. NCCT, Non-contrast CT; RSVC, Reversible cerebral vasoconstriction syndrome; CTA, Computed tomography angiography; MRA, Magnetic resonance angiography; LP, Lumbar puncture; IIH, Idiopathic intracranial hypertension.

In conclusion, we propose a clinical pathway for pediatric patients with headache in an emergency setting (Figure 1) that can guide the ED pediatrician in clinical practice.

## Author Contributions

UR, ND, and PP formulated the original idea and the design of the review and wrote the first draft of the manuscript. CO, MP, MV, and AR approved the design of the study. All authors reviewed, approved, and agreed to be accountable for all aspects of the work.

### Conflict of Interest Statement

The authors declare that the research was conducted in the absence of any commercial or financial relationships that could be construed as a potential conflict of interest. The reviewer LP declared a shared affiliation, though no other collaboration, with several of the authors UR, CO, and AR to the handling editor.
